# Characterization and Clinical Implications of Multidrug-Resistant Klebsiella pneumoniae in ICU Patients in India: A Comprehensive Review

**DOI:** 10.7759/cureus.108593

**Published:** 2026-05-10

**Authors:** Geeta Karande, Shivaji T Mohite, Satish Karande, Satish Patil

**Affiliations:** 1 Department of Microbiology, Krishna Institute of Medical Sciences, Krishna Vishwa Vidyapeeth (Deemed to be University), Karad, IND; 2 Department of Otolaryngology, Krishna Institute of Medical Sciences, Krishna Vishwa Vidyapeeth (Deemed to be University), Karad, IND

**Keywords:** antimicrobial resistance carbapenem resistance, icu infections, india, infection control, multidrug-resistant klebsiella pneumoniae

## Abstract

Multidrug-resistant (MDR) *Klebsiella pneumoniae* has become a prominent cause of healthcare-associated infections, especially in Intensive Care Units (ICUs), constituting a major threat to patient outcomes and healthcare systems.

This review examines the epidemiology, risk factors, mechanisms of antimicrobial resistance, virulence determinants, clinical manifestations, treatment approaches, infection control measures, and future perspectives of MDR *K. pneumoniae*, with particular emphasis on the Indian context.

The rising incidence of carbapenem-resistant and hypervirulent strains has severely restricted available therapeutic choices, leading to higher mortality rates, prolonged hospital stays, and increased healthcare costs. Resistance is largely driven by mechanisms such as extended-spectrum β-lactamase (ESBL) and carbapenemase production, along with plasmid-mediated gene transfer, which facilitates rapid spread. Furthermore, virulence determinants such as capsule production, siderophore systems, and biofilm formation contribute to increased pathogenicity.

Treatment remains difficult because of the scarcity of effective antibiotics, often requiring combination therapies and the investigation of novel treatment modalities. Reinforcing infection control practices, antimicrobial stewardship, and surveillance systems is crucial to curb the spread of MDR strains. Additionally, emerging approaches such as rapid diagnostic tools, genomic surveillance, and artificial intelligence-based predictive models offer potential for early detection and improved clinical outcomes.

## Introduction and background

Multidrug-resistant (MDR) *Klebsiella pneumoniae* has become a significant cause of healthcare-associated infections in Intensive Care Units (ICUs), especially in India. This Gram-negative opportunistic pathogen is commonly implicated in severe clinical conditions such as ventilator-associated pneumonia (VAP), bloodstream infections (BSIs), and urinary tract infections (UTIs) in severely ill patients. The burden is notably elevated in ICU settings because of factors including prolonged hospital stay, frequent use of invasive devices, and widespread administration of broad-spectrum antibiotics, all of which promote colonization and infection by resistant strains. Recent research from India has recognized *K. pneumoniae* as one of the leading pathogens linked to ICU-acquired infections, with rising antimicrobial resistance trends further complicating clinical management [1].

The emergence of MDR *K. pneumoniae* is primarily driven by the acquisition of diverse resistance mechanisms, notably extended-spectrum β-lactamases (ESBLs) and carbapenemases such as New Delhi metallo-β-lactamase (NDM) and OXA-48. It is essential to quantify the extent of the reduction in antibiotic effectiveness and to determine whether this decline is statistically significant. The production of these enzymes substantially compromises the efficacy of commonly used antibiotics, particularly third-generation cephalosporins and carbapenems. Molecular studies conducted in India have reported a high prevalence of carbapenem-resistant *K. pneumoniae* (CRKP) in ICU settings, reflecting extensive dissemination of resistance determinants across healthcare institutions. These observations underscore the urgent need for continuous surveillance and molecular epidemiological studies to monitor resistance trends and inform appropriate therapeutic strategies [2].

The clinical impact of MDR *K. pneumoniae* infections is considerable, leading to higher morbidity and mortality, extended hospital stays, and elevated healthcare costs. The scarcity of effective treatment options often compels the use of last-resort agents such as colistin and tigecycline, which are linked to potential toxicity and inconsistent clinical outcomes. In the Indian setting, the increasing prevalence of extensively drug-resistant strains further complicates therapeutic management and highlights the pressing need for enhanced antimicrobial stewardship and stringent infection control measures in ICUs. Tackling this escalating issue necessitates coordinated efforts in surveillance, early diagnosis, and the advancement of new antimicrobial treatments [3].

## Review

Search strategy

A comprehensive literature search was conducted using databases such as PubMed, Scopus, Web of Science, and Google Scholar to identify relevant studies. Keywords including “Klebsiella pneumoniae,” “multidrug-resistant,” “MDR,” “ICU,” and “clinical outcomes” were combined using Boolean operators (AND, OR), and appropriate MeSH terms were applied where applicable. Filters such as English language, human studies, and publication within the last 5-10 years were used to refine the results. Titles and abstracts were screened for relevance, followed by full-text assessment. Studies on MDR *K. pneumoniae* in human clinical settings reporting resistance patterns, mechanisms, or treatment outcomes were included. Non-clinical studies, articles not specific to *K. pneumoniae*, case reports, editorials, and studies with insufficient data were excluded.

Epidemiology of MDR *K. pneumoniae*

The worldwide impact of MDR *K. pneumoniae* is steadily increasing, with notable regional variations in prevalence. In Southeast Asia, including India, studies have reported a high proportion of MDR isolates, often exceeding 50%, reflecting widespread antimicrobial misuse and inadequate infection control practices. Surveillance data from India indicate that *K. pneumoniae* remains one of the most commonly isolated Gram-negative pathogens in hospital settings, particularly among critically ill patients, with a growing proportion exhibiting resistance to multiple antibiotic classes [4]. In ICUs, the burden of MDR *K. pneumoniae* infections is particularly concerning, with infection rates reported at approximately 6%-9% among ICU patients. These settings act as major reservoirs for transmission because of factors such as high antibiotic selection pressure, extensive use of invasive devices, and increased risk of cross-transmission via healthcare personnel. Critically ill patients, those with prolonged hospitalization, and immunocompromised individuals are at higher risk of infection [5]. The clinical burden is substantial, with mortality rates reaching 40%-50% in severe infections, especially BSIs and VAP. The emergence of CRKP, particularly strains producing NDM-1, further complicates treatment and highlights the rapid spread of highly resistant pathogens in hospital settings. These findings underscore the urgent need for strengthened antimicrobial stewardship, continuous surveillance, and strict infection prevention and control measures [6].

Risk factors in ICU patients

MDR *K. pneumoniae* infections in ICU patients are affected by a range of patient-related factors that enhance susceptibility. Advanced age is a well-established risk factor because of the age-associated decline in immune function, while comorbid conditions such as diabetes mellitus, malignancy, and chronic kidney disease further impair host defenses. Additionally, immunosuppression, arising either from the underlying disease state or from treatments such as corticosteroids and chemotherapy, substantially increases the risk of colonization and subsequent infection with MDR organisms [7].

Hospital-associated factors significantly contribute to the acquisition and spread of MDR *K. pneumoniae* in ICU settings. The use of invasive devices, including mechanical ventilation and central venous catheters, compromises natural host barriers and facilitates bacterial entry. Extended ICU stay increases exposure to resistant organisms, while prior antibiotic therapy, particularly with carbapenems and broad-spectrum cephalosporins, creates selective pressure that drives the emergence and persistence of resistant strains [8].

Colonization, particularly within the gastrointestinal tract, is regarded as a key precursor to infection. Studies from Indian healthcare settings have demonstrated that patients colonized with MDR* K. pneumoniae* are at a substantially higher risk of developing invasive infections, including BSIs and VAP. Early detection, targeted surveillance, and strict infection prevention and control measures are essential to limit progression from colonization to infection in high-risk ICU patients [9].

Mechanisms of antimicrobial resistance

MDR *K. pneumoniae* demonstrates a broad range of resistance mechanisms that markedly restrict therapeutic options, especially in ICU settings. These mechanisms include enzymatic inactivation by β-lactamases such as ESBLs (e.g., CTX-M) and carbapenemases (e.g., NDM, KPC, OXA-48); alterations in target sites, such as mutations in porin proteins (OmpK35/OmpK36) and penicillin-binding proteins; overexpression of efflux pumps (e.g., AcrAB-TolC); and horizontal gene transfer mediated by plasmids carrying resistance genes. Research from India has reported a high prevalence of both β-lactam and non-β-lactam resistance determinants, indicating substantial antimicrobial pressure in healthcare settings and the rapid evolution of resistance among clinical isolates [9].

β-Lactam Resistance

Resistance to β-lactam antibiotics in *K. pneumoniae* is mainly mediated through the production of β-lactamase enzymes. ESBLs, such as CTX-M, TEM, and SHV variants, are capable of hydrolyzing penicillins and cephalosporins, thereby reducing their efficacy. Of greater concern is the emergence of carbapenemases, including KPC, NDM, and OXA-48-like enzymes, which impart resistance to carbapenems, agents often reserved as last-line therapy. Reports from India have documented the extensive dissemination of NDM and OXA-48-like enzymes among clinical isolates, significantly contributing to the increasing burden of CRKP [10].

Non-β-Lactam Resistance

In addition to β-lactam resistance, *K. pneumoniae* has developed resistance to several other classes of antibiotics. Resistance to aminoglycosides is typically mediated by aminoglycoside-modifying enzymes that inactivate these drugs. Fluoroquinolone resistance arises due to mutations in target genes such as gyrA and parC, which reduce drug-binding affinity. Moreover, efflux pumps - particularly the AcrAB-TolC system - play a significant role by actively exporting antibiotics out of bacterial cells, thereby lowering intracellular drug concentrations. The interplay of these mechanisms frequently leads to MDR or extensively drug-resistant phenotypes among ICU isolates [11].

Genetic Basis of Resistance

The rapid dissemination of antimicrobial resistance in *K. pneumoniae* is largely driven by mobile genetic elements. Plasmid-mediated gene transfer facilitates the horizontal spread of resistance genes across diverse bacterial species. Transposons and integrons play a crucial role in capturing and integrating multiple resistance determinants within a single genetic platform. In addition, clonal expansion of high-risk lineages, such as sequence type ST11, has been linked to widespread hospital outbreaks. ST11 is a high-risk clone of *K. pneumoniae* strongly associated with multidrug resistance and hospital outbreaks. It commonly carries carbapenemase genes (e.g., KPC, NDM), facilitates rapid spread via plasmids, and accounts for a significant proportion of carbapenem-resistant isolates, often leading to poor clinical outcomes. Whole-genome sequencing studies, including those from India, have demonstrated substantial genetic diversity and highlighted the rapid evolution and dissemination of key resistance determinants, particularly carbapenemase genes (blaNDM, blaKPC, blaOXA-48-like) and ESBL genes (blaCTX-M), among *K. pneumoniae* strains [12].

Virulence factors

MDR *K. pneumoniae* is increasingly acknowledged not only for its resistance to multiple antimicrobial agents but also for its heightened virulence, which contributes to severe and often fatal infections in ICU patients. The combination of antimicrobial resistance and virulence factors has resulted in the emergence of highly pathogenic strains capable of causing invasive conditions such as liver abscess, septicemia, and pneumonia. Research from India has indicated a rising occurrence of these strains in hospital settings, posing significant clinical challenges [13].

One of the key virulence determinants is the capsule (K antigen), which protects the bacterium against host immune defenses by inhibiting phagocytosis and complement-mediated killing. Strains possessing a thick capsule are more capable of surviving in the bloodstream and causing systemic infections. Furthermore, *K. pneumoniae* produces siderophores such as enterobactin and yersiniabactin, which enable efficient iron acquisition from the host, thereby supporting bacterial growth and persistence in iron-limited environments such as human tissues [14].

Another important virulence factor is the presence of hypermucoviscosity-associated genes, such as *rmpA* and *rmpA2*, which regulate capsule synthesis and contribute to the hypermucoviscous phenotype. This phenotype is linked to enhanced invasiveness and an increased ability to cause severe community-acquired infections. In addition, biofilm formation on medical devices, including catheters and ventilators, promotes bacterial persistence, confers protection against antimicrobial agents, and contributes to chronic infections. This biofilm-forming capability is particularly significant in ICU settings, where the use of invasive devices is common [15].

The emergence of carbapenem-resistant hypervirulent *K. pneumoniae* (CR-hvKP) strains represents a significant public health concern, as these organisms combine extensive antimicrobial resistance with enhanced virulence. Recent reports from India have indicated an increasing prevalence of such strains, which are linked with high mortality rates and severely limited therapeutic options. This dual challenge highlights the pressing need for ongoing monitoring, prompt detection, and strengthened infection prevention and control measures to limit their spread within healthcare settings [16].

Clinical manifestations in ICU

MDR *K. pneumoniae* is a prominent cause of healthcare-associated infections in ICUs, resulting in a broad range of clinical manifestations. These infections frequently become severe because of the debilitated state of ICU patients and the organism’s capacity to resist multiple antimicrobial agents. In India, *K. pneumoniae* is a leading cause of ICU-acquired infections, associated with increased morbidity, mortality, and prolonged hospital stay [17]. Among the most frequent clinical presentations is VAP, in which *K. pneumoniae* colonizes the lower respiratory tract of mechanically ventilated patients, resulting in severe pneumonia.

MDR *K. pneumoniae* causes a range of severe infections in ICU patients, most notably pneumonia, particularly VAP, which is associated with high mortality rates of approximately 30%-50%. BSIs, often related to invasive devices such as central venous catheters, are also linked to mortality rates approaching 40%-50%. UTIs are frequently observed in catheterized patients, while surgical site infections (SSIs) occur in postoperative ICU patients, especially those with prolonged hospitalization or prior antibiotic exposure [18].

Polymicrobial infections are frequently encountered in ICU settings, where *K. pneumoniae* co-exists with other pathogens such as *Acinetobacter baumannii* and *Pseudomonas aeruginosa*. These mixed infections complicate both diagnosis and management, often resulting in inappropriate empirical therapy and unfavorable clinical outcomes. Surveillance data from India highlight the growing complexity of ICU infections due to such polymicrobial involvement, underscoring the importance of timely microbiological diagnosis and the use of targeted antimicrobial therapy [19].

Treatment strategies

Management of MDR *K. pneumoniae* infections in ICU patients is highly challenging due to limited effective antimicrobial options and the severity of illness in affected individuals. A decade-long analysis from a tertiary care hospital in India showed a rising trend of MDR *K. pneumoniae* BSIs, emphasizing increasing resistance to commonly used antibiotics and the growing need for last-resort and combination therapies. However, therapeutic outcomes remain suboptimal, emphasizing the need for individualized, evidence-based treatment guided by antimicrobial susceptibility testing and optimized through pharmacokinetic and pharmacodynamic (PK/PD) considerations, particularly in critically ill ICU patients [20].

Antibiotic Therapy

Therapeutic options for MDR *K. pneumoniae* are often limited to a small number of agents, including colistin, tigecycline, and fosfomycin. Colistin (polymyxin E) is commonly employed for the treatment of carbapenem-resistant strains, although its use is associated with concerns of nephrotoxicity and the emergence of resistance. Tigecycline exhibits activity against a wide spectrum of MDR organisms; however, its utility in BSIs is restricted due to low serum concentrations. Fosfomycin is mainly used for uncomplicated UTIs, but in MDR *K. pneumoniae* infections, it may be considered as part of combination therapy - particularly in complicated UTIs, BSIs, or other severe infections - based on susceptibility results, given its activity against certain resistant strains and good tissue penetration​​​​​​ [21].

Combination Therapy

Combination therapy is commonly utilized to improve antimicrobial efficacy and reduce the risk of further resistance development. Carbapenem-colistin and tigecycline-based regimens have been frequently used in Indian ICUs; however, current evidence and guidelines question their routine use due to limited efficacy and toxicity, recommending their use only when more effective agents are unavailable and when guided by susceptibility and PK/PD considerations. Available evidence suggests that combination therapy may offer better clinical outcomes compared to monotherapy, particularly in severe infections such as BSIs and VAP. Nevertheless, treatment success often remains suboptimal in critically ill patients due to factors such as delayed initiation of appropriate therapy, high bacterial burden, and the presence of underlying comorbid conditions [22].

Novel Therapies

Therapeutic selection for MDR *K. pneumoniae* should be guided by the underlying resistance mechanism and clinical context. β-lactam/β-lactamase inhibitor combinations, such as ceftazidime-avibactam, are preferred for KPC- and OXA-48-producing strains, while NDM-producing isolates require combination approaches such as aztreonam with ceftazidime-avibactam or agents like cefiderocol. Tigecycline and fosfomycin are typically used as part of combination regimens in selected infections, whereas colistin is increasingly reserved as salvage therapy due to toxicity concerns. Emerging strategies, including bacteriophage therapy and antimicrobial peptides, remain investigational but may offer future options for extensively drug-resistant infections [23].

Infection control and prevention

Effective infection prevention and control measures are essential for limiting the spread of MDR *K. pneumoniae* in ICUs. These environments are regarded as critical control points due to the heightened risk of transmission, driven by the presence of critically ill patients, frequent use of invasive procedures, and extensive antibiotic exposure. Research from India has shown that rigorous compliance with infection control protocols considerably lowers the occurrence of healthcare-associated infections and limits the spread of resistant organisms [24].

Hand hygiene remains the cornerstone of infection prevention. Appropriate handwashing or the use of alcohol-based hand rubs by healthcare workers is the most effective strategy to prevent cross-transmission of MDR pathogens. Contact precautions, including the use of gloves and gowns, along with isolation of infected or colonized patients, further aid in reducing both direct and indirect transmission within ICU settings. These practices are strongly endorsed in Indian infection control guidelines and have demonstrated a measurable impact in lowering infection rates [25].

Active surveillance cultures may be considered in high-risk settings such as ICUs during outbreaks, in units with a high prevalence of MDR organisms, or for screening high-risk patients (e.g., those with prolonged hospitalization or prior antibiotic exposure). Detection of colonization should prompt strict infection prevention measures, including contact precautions and cohorting, rather than initiation of antibiotic therapy unless there is clinical evidence of infection. Additionally, antimicrobial stewardship programs (ASPs) play a critical role in optimizing antibiotic utilization, minimizing unnecessary exposure to broad-spectrum agents, and curbing the development of resistance. Hospitals in India that have adopted structured ASPs have reported improved prescribing practices and a reduction in resistance trends.

Collectively, these strategies - including antimicrobial stewardship, infection prevention, molecular surveillance, and rapid diagnostic technologies (e.g., PCR-based assays and whole-genome sequencing) - constitute a comprehensive approach to controlling MDR *K. pneumoniae* in ICU settings [26].

Clinical implications

MDR *K. pneumoniae* infections in ICU settings are associated with a significant clinical and economic burden. In India, rising prevalence has been linked to increased disease severity, particularly among critically ill patients, and challenges in the timely initiation of effective therapy due to limited treatment options [27]. These infections are associated with high mortality, especially in BSIs and VAP, as well as prolonged ICU and hospital stays, increasing the risk of complications [28]. Additionally, they contribute to substantial healthcare costs due to extended hospitalization, use of last-resort antibiotics, and intensified diagnostic and infection control measures, with treatment failure remaining a major concern.

Effective management of MDR *K. pneumoniae* requires not only optimized antimicrobial therapy but also robust infection prevention and control measures, including hand hygiene, environmental decontamination, targeted surveillance, and antimicrobial stewardship, to limit transmission and improve clinical outcomes in ICU settings [29].

Future perspectives

The increasing burden of MDR *K. pneumoniae* has emphasized the critical need for novel approaches to strengthen prevention, diagnosis, and therapeutic management. In the Indian context, where antimicrobial resistance remains a major public health challenge, future strategies are progressively oriented toward the incorporation of advanced technologies into routine clinical care. Enhancing research capacity and reinforcing the surveillance network will play a pivotal role in tackling the dynamic resistance patterns and improving overall patient outcomes [30].

One promising approach is the development of vaccines, which may offer long-term protection against *K. pneumoniae* infections, particularly in high-risk groups such as ICU patients. Concurrently, rapid diagnostic technologies, including molecular assays and point-of-care tests, are being advanced to facilitate early and precise identification of MDR pathogens, enabling prompt initiation of targeted therapy. These innovations are anticipated to substantially reduce inappropriate antibiotic use and enhance clinical outcomes [31].

Genomic surveillance, including whole-genome sequencing, enables identification of resistance determinants and tracking of high-risk clones; however, its implementation in resource-limited settings is often constrained by inadequate laboratory infrastructure, limited funding, shortage of trained personnel, and disparities between urban and rural healthcare facilities. This approach has demonstrated considerable potential in Indian studies for elucidating transmission dynamics.

In addition, personalized antimicrobial therapy, guided by patient-specific characteristics and accurate microbial identification, is emerging as a strategy to enhance treatment efficacy. Moreover, AI-based predictive models are being explored for early detection and risk stratification of MDR infections, offering promising improvements in clinical decision-making and infection control practices [32].

## Conclusions

MDR *K. pneumoniae* represents a major challenge in ICU settings, particularly in India, owing to its high levels of resistance, virulence, and associated adverse clinical outcomes. It is responsible for elevated mortality rates and longer durations of hospitalization, as well as a narrowing of effective therapeutic options. Effective management depends on early detection, appropriate targeted therapy, strict infection prevention and control measures, and robust ASPs. Future approaches, including rapid diagnostic techniques and novel therapeutic strategies, are crucial for controlling its spread and improving patient outcomes.
